# Pushing Pressure Detection Sensitivity to New Limits by Modulus‐Tunable Mechanism

**DOI:** 10.1002/advs.202403779

**Published:** 2024-07-08

**Authors:** Jing Yang, Guojiang Yuan, Yong Shen, Caili Guo, Zhibin Li, Fengling Yan, Xiaolong Chen, Lin Mei, Taihong Wang

**Affiliations:** ^1^ Department of Electrical and Electronic Engineering Southern University of Science and Technology Shenzhen Guangdong 518055 P. R. China; ^2^ School of Microelectronics Southern University of Science and Technology Shenzhen Guangdong 518055 P. R. China; ^3^ State Key Laboratory of Powder Metallurgy Central South University Changsha Hunan 410083 P. R. China

**Keywords:** compression modulus, electronic skin, intellectual manipulators, pressure sensors, ultra‐high sensitivity

## Abstract

Only microstructures are used to improve the sensitivity of iontronic pressure sensors. By modulating the compressive modulus, a breakthrough in the sensitivity of the iontronic pressure sensor is achieved. Furthermore, it allows for programmatic tailoring of sensor performance according to the requirements of different applications. Such a new strategy pushes the sensitivity up to a record‐high of 25 548.24 kPa^−1^ and expands the linear pressure range from 15 to 127 kPa. Additionally, the sensor demonstrates excellent mechanical stability over 10 000 compression‐release cycles. Based on this, a well‐controlled robotic hand that precisely tracks the pressure behavior inside a balloon to autonomously regulate the gripping angle is developed. This paves the way for the application of iontronic pressure sensors in precise sensing scenarios.

## Graphical abstract

Different from the methods of constructing surface microstructures, this work achieves the design of ultra‐high sensitivity capacitive pressure sensors and controllable adjustment of the linear response pressure range by modulating the compressive modulus of the electrodes. Furthermore, active exploration is conducted in applications such as pulse wave monitoring and tactile feedback control of robotic hands.

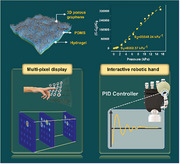

## 1 Introduction

The human tactile perception system enables an effective interaction with the external environment.^[^ ^1^ ^]^ Skin receives tactile stimuli and converts them into electrical signals, which are transmitted to the corresponding areas of the nervous system and decoded into information such as pressure and vibration.^[^ ^2^ ^]^ Consequently, humans can easily perceive sensations such as the pressure of ants crawling on the skin or the weight of a 10 kg barbell lifted in the palm of the hand. The development of flexible pressure sensing systems with skin‐like functionality offers potential applications in fields such as intelligent robotics,^[^ ^3^, ^4^, ^5^ ^]^ wearable technology,^[^ ^6^, ^7^ ^]^ and healthcare.^[^ ^8^, ^9^, ^10^ ^]^ In recent years, extensive research has focused on the development of self‐powered technologies and stretchable functional circuits.^[^ ^11^, ^12^, ^13^, ^14^ ^]^ High‐performance flexible pressure sensors combined with energy harvesting from human motion and flexible circuits for signal acquisition and wireless transmission are expected to achieve highly compact, flexible, and self‐powered intelligent sensing systems.

In recent years, the use of ionic conductors as dielectric layers has been proven to be an effective method for enhancing the performance of capacitive pressure sensors. The formation of an electric double layer (EDL) at the electrode–dielectric interface significantly increases the capacitance per unit area and effectively promotes the piezocapacitive effect during compression.^[^ ^15^, ^16^ ^]^ For this type of iontronic pressure sensor, the growth rate of the contact area at the ionic‐electronic interface under mechanical stimulation is pivotal for the sensor performance. Previous research has extensively focused on optimizing performance through surface microstructure engineering.^[^ ^15^, ^16^, ^17^, ^18^, ^19^, ^20^ ^]^ According to the Persson contact theory for randomly rough surfaces, changes in the contact area depend not only on the geometric parameters of the surface morphology but also on the effective modulus of the structure.^[^ ^21^ ^]^ A decrease in the structural stiffness of a material can effectively enhance its compressibility. Therefore, we believe that adjusting the material modulus could be another effective way to overcome the performance limitations of current iontronic pressure sensors and achieve ultra‐sensitive sensors.

**Figure 1:**
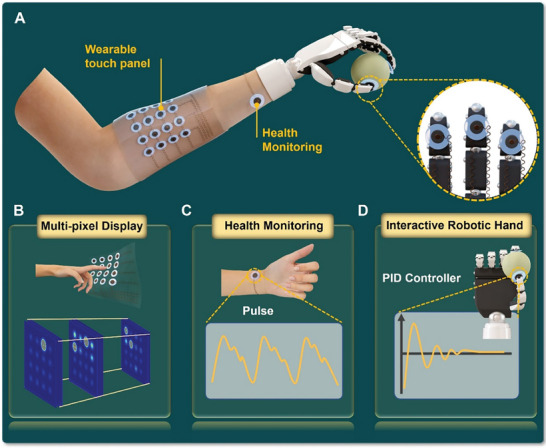
Overview of the GI pressure sensor. A) Schematic illustration for the intelligent sensory feedback skin system with GI pressure sensor integration. B) Visualization of dynamic stimuli for flexible conformal attachable GI sensing arrays. C) GI sensor for real‐time wrist pulse monitoring. D) Proportional‐integral‐derivative (PID) controlled robotic hand interaction system based on a capacitive response signal from GI pressure sensor.

Here, unlike microstructure surface engineering, we achieve an ultra‐sensitive capacitive pressure sensor with a tunable linear pressure range by adjusting the compressive modulus of the electrodes. We utilize self‐organized 3D porous graphene as the electrode. During compression loading, the graphene branches bend and undergo elastic compression. By varying the content of silver nanowires in the graphene, the Young's modulus can be effectively adjusted. We achieve an ultra‐high sensitivity of 25548.20 kPa^−1^. Furthermore, the device demonstrates excellent mechanical stability, enduring over 10000 compression/release cycles at 7 kPa without significant fatigue. We also construct a 4 × 4 flexible sensing array that can conformally adhere to the surface of a curved object for the visual display of real‐time pressure stimuli. Integrating this porous graphene‐based ionic (GI) pressure sensor with a control system enables the design of industrial robotic hands for the precise manipulation or prosthetic limbs, offering real‐time tactile feedback (as shown in **Figure** **1**).

## 2 Results

### 2.1 Fabrication and Working Mechanism

Based on the Gouy–Chapman–Stern model, for iotronic pressure sensors, when the electrode and the ionic active layer are in contact, the electronic carriers on the electrode surface have a strong electrostatic interaction with the ions in the active layer.^[^ ^22^ ^]^ Here the counter‐ion electrostatic interactions and diffusion effects follow the Poisson‐Boltzmann relationship to reach an equilibrium and produce an EDL region. In ionic pressure sensors, the variation of EDL capacitance is the key to improve the sensitivity of the device. The induced capacitance of the EDL can be expressed in the following format:

(1) $$C_{EDL}\;=\;UAC\cdot A$$

**Figure 2:**
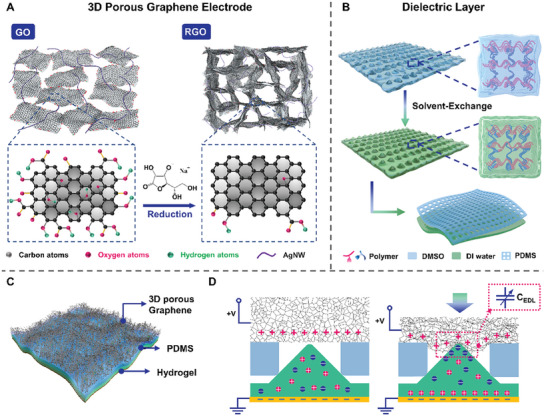
Design and preparation of the GI pressure sensor. A) Schematic diagram of the synthesis of 3D porous graphene electrode. B) Schematic structure of ionic dielectric layer. The lower layer is a PVA hydrogel with pyramidal microstructure prepared by solvent substitution strategy. The upper layer is a porous PDMS membrane. C) Design architecture of the GI pressure sensor composed of 3D porous Graphene, porous PDMS, ionic hydrogel, and Cu electrode. D) Sensing principles of the GI pressure sensor.

UAC is the capacitance per unit area and depends mainly on the type and concentration of ions in the ionic dielectric layer. For a given system, the UAC can be simplified and analyzed as a constant. A is the contact area between the electrode layer and the ionic dielectric layer. Therefore, the change in contact area A when pressure is applied is the key to causing a change in EDL capacitance and improving sensitivity. According to the Persson contact theory for random rough surfaces, the normalized contact area can be expressed as:^[^ ^21^, ^23^ ^]^

(2) $$\frac{\Delta A}{A_{0}}=\frac{A-A_{0}}{A_{0}}\;=\;\varphi \frac{P}{E}$$

where A_0_ is the initial contact area, ∆A = A‐A_0_ is the change in contact area under applied pressure. φ is a geometric parameter dependent on the surface morphology, P is the applied stress, and E is the effective modulus of elasticity of the surface. Therefore, the design of the device geometry as well as the modulus of elasticity is crucial to regulate the device performance. Self‐assembled 3D porous graphene has a rich surface morphology (φ), which helps achieve a larger contact area under saturated pressure. More importantly, the porous structure of graphene significantly reduces the compressive modulus (E) of the electrode, allowing for greater deformation under pressure. This boosts the contact area growth rate at the ion‐electrode interface during compression, significantly enhancing sensor sensitivity. Based on the above analysis, we believe that graphene with a 3D porous network structure is an excellent choice of electrode for realizing high‐sensitivity iontronic pressure sensors. **Figure** **2**A demonstrates the mechanism of self‐assembly of graphene oxide (GO) lamellae to form 3D porous graphene electrodes. The surface of commercial GO lamellae uniformly dispersed in water has many oxygen‐containing groups. In the presence of sodium ascorbate, the number of oxygen‐containing groups decreases. GO changes from hydrophilic to hydrophobic and self‐assembles to form a 3D structure (shown in Figure S1, Supporting Information). And graphene electrodes with 3D porous network structure were obtained after freeze–drying. The exact fabrication process is described in detail in the experimental section. Based on this scheme it is possible to prepare graphene electrodes of large sizes with different shapes or on a large scale as needed. Due to the rich 3D porous network structure of the graphene electrode obtained by self‐assembly, a large number of positive and negative ion pairs are distributed at the interface between the porous electrode and the ionic dielectric layer when fully compressed. Compared with the flat electrodes, porous electrodes have a larger surface area for the formation of EDL interface. Therefore, the sensitivity of the GI device can be effectively improved. In addition, we added different ratios of silver nanowires (AgNW) to GO aqueous solution and found that it can be well self‐assembled with graphene to obtain a 3D network structure. And the Young's modulus of 3D porous graphene electrode can be regulated by controlling different AgNW contents. Based on our previous analysis, the design of the elastic modulus of the electrode material also plays a crucial role in regulating the performance of the device. Here we fabricate different graphene electrodes with AgNW mass fractions of 0 (0A‐G), 10% (10A‐G), and 20% (20A‐G) respectively for experimental demonstration.

After applying an applied voltage, the electrons on the top and bottom electrodes attract ions with opposite polarity in the ionic dielectric layer to migrate and accumulate at the contact interface of the electrode/dielectric layer to form EDL with ultra‐high capacitance is the key to iontronic pressure sensors. Here we constructed a polyvinyl alcohol (PVA) hydrogel containing sodium chloride ions by a simple and efficient solvent exchange strategy.^[^ ^24^ ^]^ The fabrication process of the hydrogel is shown in Figure 2B and Figure S2 (Supporting Information). First, we machined inverted conical arrays with a depth of 0.4 mm, a diameter of 4 mm, and a spacing of 1 mm on the surface of polytetrafluoroethylene (PTFE) material as microstructural templates for hydrogels using a CNC machine. The homogeneous solution of PVA dissolved in dimethyl sulfoxide (DMSO) is poured onto the microstructure template. It is then completely immersed in NaCl aqueous solution, accompanied by the occurrence of solvent substitution (NaCl aqueous solution replacing DMSO) to obtain NaCl‐PVA hydrogel. Here DMSO acts as a strong hydrogen bonding acceptor and is a good solvent for polyvinyl alcohol (PVA). The formation of polymer‐solvent hydrogen bonds is easier in the initial solution. The use of a relatively poor solvent (NaCl aqueous solution) during the crosslinking step restores intra‐ and inter‐polymer hydrogen bonding within the PVA polymer to form a tough hydrogel network. Therefore, the resulting NaCl‐PVA hydrogels have significant mechanical strength. To validate the synchronous entry of NaCl ions into the hydrogel network during the solvent exchange process, we characterize the NaCl‐PVA hydrogel after freeze–drying. As shown in Figure S3A,B (Supporting Information), scanning electron microscope (SEM) images of the surface and cross‐section of the hydrogel after freeze–drying respectively revealed abundant precipitated particles within the dried hydrogel network. Energy‐dispersive X‐ray spectroscopy characterization is conducted at position Figure S3A (Supporting Information), and the elemental distribution maps obtained from the scans (Figure S3C, Supporting Information) show the presence of Na and Cl elements corresponding to the locations of the precipitated particles, indicating the formation of sodium chloride crystals. This also confirms that NaCl ions indeed entered the hydrogel network during the formation process via solvent exchange. In addition, we prepared a through‐hole PDMS spacer with a diameter of 4 mm and a pitch of 1 mm using laser ablation. The detailed fabrication process is described in detail in Figure S4 (Supporting Information) and in the experimental section. The position of each through‐hole on the PDMS spacer corresponded to the position of the microcones of the NaCl‐PVA hydrogel. Since the elastic modulus of PDMS is higher than that of NaCl–PVA hydrogel, this PDMS spacer can be used as a support frame. On the one hand, it can avoid the contact between the ionic layer and the electrode layer at the initial stage, ensuring consistent initial capacitance across different batches and avoiding early EDL formation. On the other hand, it also facilitates the rapid recovery of the device after the elimination of external mechanical stimuli and mitigates the unfavorable effect of viscoelastic hysteresis. The compressive stress–strain curves of PDMS and NaCl‐PVA hydrogel are shown in Figure S5 (Supporting Information). The results show that the elastic modulus of the experimentally prepared PDMS is about 1.87 MPa, and the elastic modulus of NaCl‐PVA hydrogel is about 122.39 kPa.

The schematic of the hierarchical structure of the constructed GI pressure sensor is shown in Figure 2C. The upper layer is a porous graphene electrode formed by self‐assembly. By adjusting the content of silver nanowires in graphene we prepared three sets of porous graphene electrodes with different modulus. The second layer is a through‐hole PDMS spacer layer. The third layer is the active layer of NaCl–PVA hydrogel with microcone structure prepared by solvent‐exchange method. The bottom layer is a commercially procured PI–Cu electrode. In the absence of pressure stimulation, there is an air spacing between the top graphene electrode and the ion‐active layer due to the presence of the spacer layer, at which time no EDL is formed at the interface between the top electrode and the ion layer (shown on the left in Figure 2D). Under pressure stimulation, porous graphene with low Young's modulus deforms and comes into contact with the ionic hydrogel layer in the spacer layer through‐holes. Positive and negative charges on the two electrode layers under the action of an applied electric field attract anions and cations of opposite polarity on the NaCl‐PVA hydrogel layer, respectively. These electron‐ion pairs form a capacitor at the nanometer separation distance scale, which can result in ultra‐high capacitance per unit area. As the pressure continued to increase, the contact area between the ion‐active layer and the graphene electrode layer further increased. Based on the previous EDL model, the increase of the contact area (A) can effectively increase the EDL capacitance value of the device.

**Figure 3:**
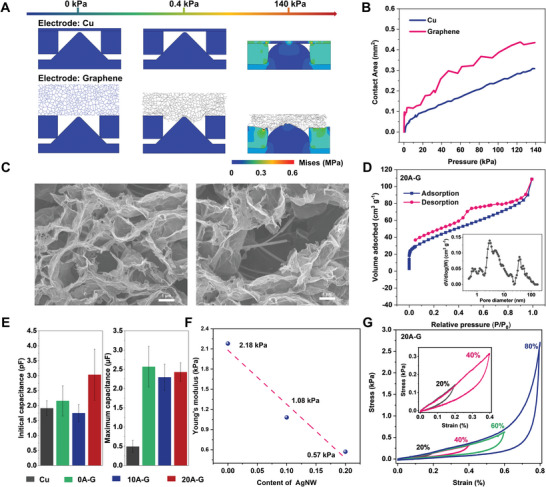
Sensing principles of GI pressure sensor and the properties of its 3D porous graphene. A) Simulation results of stress distribution in graphene (0A‐G) and Cu electrodes at pressures up to 140 kPa. B) Variation of the contact area between the dielectric layer and two different opposing electrodes over a sensing range of 140 kPa. C) SEM images of 20A‐G at different magnifications. D) Nitrogen adsorption/desorption isotherms and the related pore size distribution of the 20A‐G. E) initial (*C*_0_, without pressure) and final (*C*_p_, with pressure of 127 kPa) of the prepared ionic pressure sensor with various electrode. F) Young's stress–strain versus AgNW content of porous graphene. G) Compressive stress–strain curves of 20A‐G during compression‐release cycles with maximum strains up to 80%.

We comparatively studied the compression process of copper electrodes with high modulus and graphene electrodes with 3D pore network structure to elucidate the deformation response mechanism of the device after pressure stimulation. The schematic diagrams of the different compression stages of the device by finite element analysis (FEA) is shown in **Figure** **3**A. Due to the fact that 3D full‐size simulation requires a large number of computational resources, based on the symmetry of the device structure, we simplified the FEA process by selecting only one microstructural unit of the device for simulation. To demonstrate the validity of the model, we compare the constructed graphene model and the graphene samples in terms of overall density and mechanical properties. The density of the porous graphene model is 6.6 kg/m^3^, while the experimental sample density is 6.64 kg m^−^^3^, with a discrepancy of less than 1%. Additionally, we compare the uniaxial compression simulation results of the graphene model with the test results of the graphene samples. As shown in Figure S6 (Supporting Information), the Young's modulus of the graphene model is 2.27 kPa, while the experimental sample's Young's modulus is 2.18 kPa, with a discrepancy of less than 5%. Therefore, we believe the porous graphene model we established is reasonable. As can be seen from the simulated deformation diagrams, due to the high modulus of the PI–Cu electrode, the electrode as a whole moves downward under pressure stimulation, compressing the PDMS spacer layer and the NaCl–PVA hydrogel layer to deform. This causes a change in the contact area of the electrode and ionic hydrogel layer. Unlike the deformation process of PI–Cu electrodes, graphene electrodes exhibit an overall ultra‐low Young's modulus and excellent compressibility due to their rich porous network structure. Thus, the deformation response mechanism of GI sensors under pressure stimulation can be divided into two stages. First the porous graphene electrode deforms under pressure and comes into contact with the ionic hydrogel layer located in the through holes of the spacer layer. The contact interface between the graphene electrode and the hydrogel induces a rapid increase in the device capacitance due to the formation of the EDL. As the pressure increases, the hydrogel with a microcone structure also deforms during compression, which further causes an increase in the area of the EDL. And due to the rich 3D porous network structure of graphene electrode, it has a higher specific surface area compared to PI–Cu electrode. Thus, fully compressed case graphene electrode‐based ionic pressure sensors have a larger EDL area. Unlike graphene electrodes, PI–Cu electrodes are deformed by extruding the PDMS spacer layer due to the higher Young's modulus and the downward shift of PI–Cu as a whole during initial compression. At this stage, mainly the air gap layer generated by the spacer layer is squeezed, and the device capacitance changes are small. As the intensity of the external mechanical stimulus increases, the device is further squeezed, and contact occurs between the PI–Cu electrode and the ionic hydrogel layer, creating an EDL capacitance at the interface. As the intensity of the mechanical stimulus increases, the device is further squeezed. The compressive deformation of the hydrogel layer can effectively increase the area of the EDL, resulting in a sharp increase in the response capacitance of the device. Figure 3B shows the simulation results of the graphene electrode, and PI–Cu electrode and ionic hydrogel layer contact area variation with pressure. The results show that the graphene electrode has a larger contact area at a given pressure, and the change in contact area of the graphene electrode is more significant at small pressures. Based on the previous analysis of the sensing mechanism, when the electrode and the ionic hydrogel layer are in contact, a large number of positive and negative ion pairs are distributed at the interface, and the signal amplitude at the interface is determined by the contact area.

We also characterize the morphology, pore structure, and mechanical properties of graphene electrodes. Figure 3C shows the SEM images of 20A‐G at different magnifications. The 3D porous network structure formed by the assembly of graphene lamellae can be clearly seen in the SEM image. It can also be observed that the silver nanowires added in the precursor solution are successfully embedded in the graphene porous network formed by self‐assembly. Simultaneously, no significant aggregation of AgNW is observed under the SEM. Our previous work also confirms that AgNW can be uniformly embedded within the 3D porous graphene during the self‐assembly process.^[^ ^25^ ^]^ Porous structure and specific surface area of 3D porous graphene electrodes obtained from nitrogen adsorption–desorption isotherms, as depicted in Figure 3D and Figure S6 (Supporting Information). N_2_ adsorption measurements on porous graphene show typical typeIIadsorption isotherm characteristics and H_4_ hysteresis loops. The specific surface area calculated from the Brunauer–Emmett–Teller (BET) equation is 172.9412 m^2^g^−1^ (0A‐G), 106.9006 m^2^g^−1^ (10A‐G), 147.3229 m^2^g^−1^ (20A‐G), respectively. The pore sizes in the 3D porous graphene electrodes are mainly concentrated in the mesopore and macroporous scale range. These pores well improve the compressibility of graphene electrodes. It can also be noticed that the addition of silver nanowires has no significant effect on the specific surface area and pore size distribution of the electrode. As shown in Figure 3E, the capacitance values of ionic hydrogel pressure sensors with four different electrodes at 0 and 127 kPa are demonstrated, respectively. Thanks to the air gap created by the though‐hole PDMS spacer layer, the initial capacitance values of the four devices are very small all in the order of picofarads. The capacitance of the device made by PI‐Cu electrodes without microstructures on the surface under external pressure stimulation at 127 kPa is only about 500 nF. However, the capacitance values of the ionic pressure sensors prepared with 3D graphene electrodes with different silver nanowire contents can reach microfarads. This is mainly due to the ultra‐high specific surface area brought by the rich porous structure of graphene electrodes, which effectively increases the area of the EDL capacitance of the device. As indicated by the BET results, the addition of AgNW has a small effect on the specific surface area and pore structure of graphene electrodes, thus exhibiting no significant difference in the capacitance values exhibited by graphene ionic pressure sensors with different AgNW contents under the stimulation of saturation pressure (127 kPa).

The content of AgNW mainly has a significant effect on the Young's modulus of graphene electrode. We investigated the mechanical properties of graphene electrodes by quasi‐static uniaxial compression release tests. The samples are subjected to controlled compressive stress on a compression testing machine, while simultaneously recording the stress–strain data collected during this process. The Young's modulus of each sample is calculated from the initial linear portion of the stress–strain curve, where the material exhibited elastic behavior. The results show that the compressive modulus of pure 3D graphene electrodes(0A‐G), and composite 3D graphene electrodes with AgNW contents of 10 wt.% (10A‐G) and 20 wt.% (20A‐G) reach values as low as 2.18, 1.08, and 0.57 kPa, respectively (shown in Figure 3F). The detailed stress–strain curves for graphene with different AgNW concentrations are presented in Figures S6B and S8 (wt%). The modulus is more than 1000 times lower than that of electrodes conventionally using elastomers as a substrate.^[^ ^15^ ^]^ This allows the graphene electrode to deform under small pressure stimuli, thereby increasing the contact area between the electrode and the ionic hydrogel and generating a larger response signal. It can also be noted that the Young's modulus of graphene electrode has a good linear correlation with the content of AgNW. It is nearly three times lower than the initial AgNW‐free sample when the addition of AgNW reaches 20 wt.%. In addition, the elastic properties of monolithic graphene electrodes are characterized during uniaxial compression‐release cycles. A low residual strain (<1%) is observed after the first compression release cycle with a maximum strain of 80% (shown in Figure 3G; Figure S9, Supporting Information). The 3D graphene primarily relies on the bending and elastic compression of its branches.^[^ ^26^, ^27^, ^28^, ^29^ ^]^ It is also worth noting that the 3D porous graphene electrode exhibits significant hysteresis during the compression release process. Recent literature has reported that techniques such as thermal annealing and directional freezing can further enhance the mechanical elasticity of 3D graphene.^[^ ^30^, ^31^ ^]^ In our study, we employ a high‐modulus PDMS spacer layer to achieve rapid separation between the electrodes and the ionic hydrogel layer upon pressure release. This effectively remedies the problem of long recovery time of the pressure sensor response that may be caused by the electrode strain hysteresis. We are also exploring further options to improve the hysteresis phenomenon of 3D graphene electrodes, hoping to further improve their mechanical elasticity while maintaining their low modulus.

### 2.2 Sensing Properties of the GI Pressure Sensor

**Figure 4:**
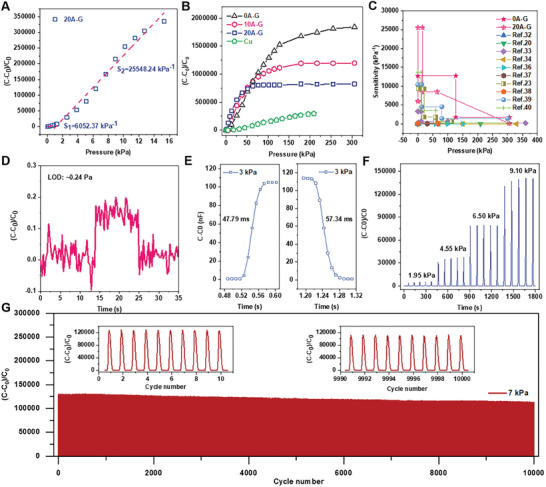
Sensing properties of the ionic pressure sensor. A) Change of capacitance over the pressure range of 15 kPa for 20A‐G ionic pressure sensor. B) Normalized change in capacitance as a function of pressure for the prepared ionic pressure sensor with different electrode. C) Performance comparisons of our pressure sensor with reported ionic sensors. D) Limit of detection (LOD) for 20A‐G pressure sensor. E) Response and recovery time of 20A‐G pressure sensor under 3 kPa transient pressure stimulus. F) Relative capacitance changes over time for 20A‐G pressure sensors at 1.35, 4.55, 6.50, and 9.10 kPa. G) Durability of the 20A‐G pressure sensor under 7 kPa.

Sensitivity and linearity are key parameters in determining the performance of pressure sensors. For capacitive type pressure sensors, the sensitivity (S) is calculated by the formula: S = *δ*(*C*‐*C*_0_)/*C*_0_. *C*_0_ is the initial capacitance value of the device when there is no external pressure loading. C is the value of the capacitance signal generated by the corresponding device when different external pressures are loaded. As previously mentioned, due to the presence of the through‐hole PDMS spacer layer, the device has a layer of air gap between the electrode and the ionic hydrogel layer at the initial stage. This gap effectively avoids the formation of an initial state EDL capacitance, which reduces the initial capacitance value of the devices. And thanks to the rich porous network structure inside the 3D graphene electrode and the ultra‐low modulus obtained by introducing AgNW, the electrode can easily deform under small pressure compression to contact with the tapered ionic hydrogel inside the PDMS pores to form an EDL. This causes a rapid increase in the response capacitance signal. **Figure** **4**A illustrates the normalized variation of the capacitance of the sensor constructed with graphene electrodes with 20% AgNW content over a small pressure range of 15 kPa. The blue points are the data values of the experimental measurement, and the red curve is the fitting result. The results show that the 20A‐G pressure sensor has a sensitivity of 6052.37 kPa^−1^ for a pressure range of <0.6 kPa, and an ultra‐high sensitivity of 25548.24 kPa^−1^ for a pressure range of 0.6‐15 kPa. Also, the response capacitance normalized curves of different batches of 20A‐G pressure sensors show a good consistency (shown in Figure S10, Supporting Information).

We use the same tapered ionic hydrogel membrane sleeved inside a through‐hole PDMS spacer layer as the sensitive active layer and select different electrodes to prepare the pressure sensors. The compared result of their sensitivity curves is demonstrated in Figure 4B. As mentioned before, the rich porous network structure of graphene electrode gives it an ultra‐high specific surface area and rich surface microstructure. The addition of AgNW does not change the special porous structure of 3D self‐assembled graphene electrodes. Therefore, compared with the PI–Cu electrode, which tends to have a smooth surface, the three sets of graphene electrodes with different AgNW content can form a larger EDL interface with ionic hydrogel under pressure stimulation. We can also see from the measurement results that the performance of the pressure sensors constructed by the three groups of graphene electrodes with different AgNW contents is much better than that of the pressure sensors constructed by the PI–Cu electrodes. As we mentioned before, the increasing AgNW content in the GO solution can effectively reduce the Young's modulus of the self‐assembled 3D porous graphene electrode. The 20A‐G electrode with low Young's modulus is more prone to deformation under small pressure compression and causes a significant increase in response capacitance. Therefore, the 20A‐G pressure sensor has higher response sensitivity in the small pressure range of 15 kPa. However, as the external pressure stimulus increases, the deformation of the low‐modulus electrode material is more likely to reach saturation. Increasing the modulus of the electrode material by reducing the AgNW content in the graphene electrode can effectively broaden the linear response range of the pressure sensor. The pure graphene electrode (0A‐G) can realize an ultra‐high sensitivity of 12734.533 kPa^−1^ over an ultra‐wide pressure range of 127 kPa and has an excellent linearity (*R* ^2^ = 0.99) within this range. The sensitivity fitting results for each pressure sensor within their respective linear response pressure ranges are detailed in Figure S11 (Supporting Information). Here we effectively modulate the sensitivity and linear pressure response range of the iontronic pressure sensor by controlling the content of AgNW to adjust the Young's modulus of the electrode material. The important modulation of the material modulus on the performance of the iontronic pressure sensor is successfully demonstrated. As shown in Figure 4C, thanks to the ultra‐high EDL capacitance formed by the porous graphene electrodes in contact with the ionic hydrogel, the pressure sensor with pure graphene electrodes has an ultra‐high sensitivity over a wide linear pressure range of 127 kPa compared to other recently published iontronic pressure sensors.^[^ ^20^, ^23^, ^32^, ^33^, ^34^, ^35^, ^36^, ^37^, ^38^, ^39^, ^40^ ^]^ The sensitivity of the device can even be doubled again if 20A‐G electrodes with even lower modulus are selected. The comparison of Specific parameters between different devices is illustrated in Table S1 (Supporting Information).

In addition, graphene‐based iontornic pressure sensors also have good resolution for objects with ultra‐small masses. As shown in Figure 4D, 20A‐G pressure sensor exhibits a low limit of detection (LOD) of 0.24 Pa. To evaluate the response and recovery time of graphene‐based ionic pressure sensors after being exposed to external mechanical stimuli, we quickly and carefully placed a 20 g weight (≈3 kPa) on top of the device for ≈1s before removing it quickly. The response and recovery times of the sensor are 47.79 ms and 67.34 ms, respectively (Figure 4E). As mentioned previously in the compressive stress–strain test results, the 3D porous graphene material has some hysteresis in the recovery process after compressive deformation. This affects the recovery time of the pressure sensor to some extent. Here, we choose PDMS silicon rubber with high Young's modulus as the skeletonized spacer layer of the device, which facilitates the rapid separation between the sensor electrodes and the ionic dielectric layer after pressure release. In the future, we also hope that we can seek a more effective way to improve the strain hysteresis phenomenon of the material on the basis of maintaining the ultra‐low Young's modulus and rich microstructure of the 3D porous graphene electrodes, and to increase its recovery rate after compression deformation, thus further improving the response and recovery time of the device. For flexible pressure sensors, high mechanical durability during long‐term or cyclic use is essential for reliable signal output. Four different pressure points (1.95, 4.55, 6.50, 9.10 kPa) are selected for each of the five compression/release cycle tests within the linear response pressure interval of the 20A‐G pressure transducer, and the response capacitance normalized curves are shown in Figure 4F. During this process, the device shows a good stability consistent with the applied stresses. To further demonstrate the durability of the graphene‐based ionic pressure sensor, 10000 repeated pressure loading and release cycle measurements are performed at a peak pressure of 7 kPa (Figure 4G). The insets of Figure 4G represent the normalized capacitance response curves for the first 10 pressure loading/release and the last 10 pressure loading‐release processes, respectively. After prolonged pressure loading‐unloading cycles, the sensor signal shows ≈10% decay, demonstrating good overall stability without signal fluctuations. Additionally, we conduct electrical reliability tests on the porous graphene electrodes (20A‐G) used in the sensor. Under a peak stress of 7 kPa, we perform continuous loading and unloading cycles, measuring the I‐V curves of the electrodes every 200 compressions. The results, shown in Figure S12 (Supporting Information), indicate that the porous graphene electrodes exhibit excellent electrical stability throughout the testing process. Electromechanical durability under long‐term cyclic pressure loading‐unloading conditions is a key factor in the robust application of pressure sensors. Based on the modulus modulation mechanism, we have successfully realized iontronic pressure sensors with ultra‐high sensitivity, adjustable linear pressure response range, and high stability to mechanical loads. This also means that our devices have greater modulation space and application potential when facing different application scenarios and performance requirements, such as health monitoring, wearable devices, and intelligent robots.

In practical applications, pressure sensors often need to operate in varying environments, making it crucial to study the effects of temperature and humidity changes on sensor performance. As shown in Figure S13A,B (Supporting Information), we place a 20 g weight on the surface of the GI pressure sensor and measure capacitance changes within temperature ranges of 25–50 °C and humidity ranges of 50%–85%. The experiments are conducted in a controlled temperature and humidity chamber. After reaching the set conditions, the sensor is left for 30 min to ensure accurate measurements. The results indicate that the sensor's capacitance significantly increases with rising temperature, while humidity changes have a minimal effect. According to the Arrhenius equation, the conductivity of the hydrogel dielectric layer increases with temperature, leading to a higher ion concentration at the electrode–dielectric interface. This results in more ions forming the EDL, thereby increasing the sensor's capacitance. In fact, the effect of temperature on the capacitive signals of capacitive pressure sensors is well‐documented. According to previous studies, the charge relaxation time (*τ* = ε/σ, where ε is the dielectric constant and σ is the ionic conductivity) is temperature‐sensitive and unaffected by the sensor's stress‐induced deformation.^[^ ^1^ ^]^ This allows for the complete decoupling of thermal and mechanical stimuli in iontronic pressure sensors. Additionally, the sensor's mechanical response can be calibrated at different temperatures and combined with a temperature sensor for accurate calibration based on the actual application environment.^[^ ^41^, ^42^ ^]^ Benefiting from its excellent mechanical flexibility, the GI pressure sensor can be attached to the surfaces of complex structures. Therefore, we conducted tests on the sensor's response signals under different bending radius. As shown in Figure S13C (Supporting Information), the relationship between normalized capacitance and applied stress of the sensor under various curvature radii is displayed. Due to the constraints of the encapsulation layer, the initial capacitance of the GI pressure sensor increases rapidly as the bending radius decreases. As sensitivity depends on the initial capacitance, it decreases during this process. However, from the relationship graph between capacitance change (Δ*C* = *C*‐*C*_0_) and pressure (Figure S13D, Supporting Information), it can be observed that the capacitance change remains stable under the same external stress, regardless of the curvature radius. To reduce frequent calibration, Δ*C* testing can be employed in practical applications to obtain corresponding stress information. Many research efforts are also focused on developing bend‐insensitive pressure sensors. By constructing stress‐sensitive rigid islands and soft matrix connections using substrate materials with significantly different elastic moduli, it is possible to effectively reduce the interference of strain on the sensor's response signals.^[^ ^43^ ^]^

### 2.3 Physiological Signal Detection

**Figure 5:**
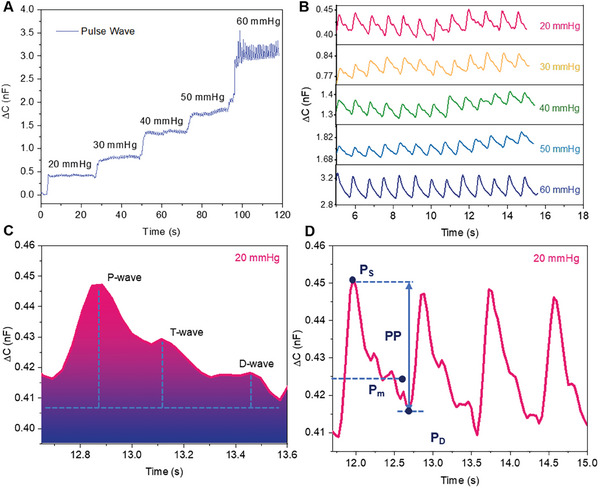
GI pressure sensor detection of wrist pulse wave signals. A) Continuous wrist pulse wave signals output by the GI pressure sensor under different preload stresses; B) Pulse waveforms detected at the wrist position under preload stresses ranging from 20 to 60 mmHg. C) Typical fingertip pulse wave signal detected under a preload of 20 mmHg. D) Feature points for K‐value calculation.

Pulse wave, as a crucial component of human physiological signals, carries abundant physiological information. Its variations can reflect the functioning status of the cardiovascular system and overall health condition to a certain extent. It holds significant importance in assessing the trend of cardiovascular diseases, predicting potential risks, and devising personalized healthcare plans. GI pressure sensors, with their ultra‐high sensitivity and rapid response to mechanical stimuli, enable the detection of minute pulse signals. We gently attached the GI pressure sensor to the position of the wrist blood vessel and secured it using the cuff of a blood pressure monitor. During the testing process, we inflated the cuff of the blood pressure monitor using its inflation pump to apply five different preload stresses ranging from 20 to 60 mmHg, maintaining each preload stress for ≈20 s. **Figure** **5**A demonstrates the variation in the response capacitance of the GI pressure sensor throughout the entire testing process. As the preload stress increases, the response capacitance of the sensor also increases. Thanks to the excellent sensitivity of the sensor, it can precisely monitor minute pulse wave signals under different preload stresses. Figure 5B displays the pulse waveforms output by the GI pressure sensor under each preload stress. The pulse wave cycles monitored without preload stress exhibit good consistency. When the preload stress reaches 60 mmHg, noticeable deformation can be observed in the pulse waveform signals, indicating that excessive preload stress interferes with the pulse. This phenomenon is consistent with observations in previous literature on pulse wave monitoring.^[^ ^44^, ^45^ ^]^ Therefore, in practical applications, pulse wave signals should be measured under a reasonable preload pressure. As shown in Figure 5C, a characteristic peak of the pulse wave signal is obtained under a preload of 20 mmHg, which allows for accurate monitoring of the P wave, T wave, and D wave in the pulse wave signal. Cardiovascular physiological and pathological changes can alter the characteristics and area of pulse wave signals. The characteristic parameter K‐value determines the degree of arterial stiffness and is a critical physiological indicator in clinical cardiovascular examinations.^[^ ^46^ ^]^ The formula for calculating the K‐value is as follows:^[^ ^47^ ^]^

(3) $$K\;=\frac{P_{m}-P_{d}}{P_{s}-P_{d}}$$

where $P_{m}=\frac{1}{T}\;\int_{0}^{T}p\left(t\right)dt$ is the mean arterial pressure, T is the duration of the cardiac cycle, *P*_s_ is the systolic peak, and *P*_d_ is the diastolic trough. The characteristic points are marked in Figure 5D. Since the K value relies on the precise measurement of pulse wave characteristics, accurate pulse wave signal monitoring is crucial for reliable evaluation. This highlights the importance of developing advanced sensor technology capable of providing high‐fidelity pulse wave data for clinical applications.

### 2.4 Detections of Static and Dynamic Subtle Motions

**Figure 6:**
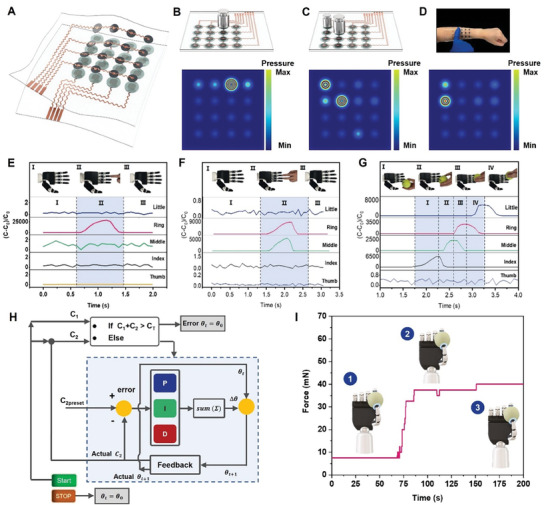
Iontronic skin for pressure visualization and its application in robotics. A) Schematic structure of GI sensor‐based iontronic skin (4×4 pixels). B,C) Visual mapping of the change in capacitance corresponding to the placement of different masses of weights at different locations on the ionic skin. D) 2D touch array with 16 capacitance‐varying visualization pixels attached to a human forearm. A capacitance change visualization with 16 pixels is shown. E–G) Changing in dynamic capacitance of GI sensors integrated in the fingers of a bionic robot during interaction with a human hand and after being rolled by a tennis ball. H) Operating schematic of a closed‐loop proportional‐integral‐derivative (PID) system for controlling a manipulator based on GI sensor pressure monitoring. I) Closed‐loop control realizes the pressure change when the manipulator lightly grips the balloon.

There is a limit to the amount of pressure information that can be detected by a single sensor because a single point of force can only produce a single capacitive response signal. Here, we propose a wearable multipixel sensing array based on GI pressure sensors. The structure of the array is schematically shown in **Figure** **6**A. Flexible and transparent PDMS films are chosen as the device encapsulation materials for both the top and bottom. The overall flexibility of the sensor array can be ensured while minimizing the damage caused by environmental factors. The PI–Cu conductive material is cut into patterned electrodes with 4 × 4 pressure sensor response sites using a laser cutter and connected to the external circuitry by serpentine leads. The upper electrode of the sensing array uses conductive silver paste to immobilize porous graphene electrodes on the cut PI–Cu response sites. The lower electrodes use the patterned PI–Cu conductive material directly. The intermediate dielectric layer is stacked with an ionic hydrogel layer with a microcone structure and a through‐hole PDMS spacer layer sequentially from bottom to top according to the structure of the GI pressure sensor. Measurement data is transferred to a computer via a universal serial bus serial port, and after processing it can be patterned in real‐time to display the capacitance difference mapping of the GI array. The detailed fabrication process is described in the experimental section. When we place an object on the GI array, the GI array outputs a significant capacitive response signal. And a color change occurs at the corresponding position in the real‐time pressure mapping map (Figure 6B). Differentiated pressure display can also be realized when placing objects with different mass, and the mapping of the output capacitance signal can correspond to the position of the object on the array (Figure 6C). Thanks to the flexible nature of the GI sensing array, it can be perfectly conformally attached to the human forearm to realize a 2D touch panel based on the iontronic principle (Figure 6D). Based on this we design a 2D wearable flexible touch panel that enables multi‐touch. It is capable of accurately detecting the position of the contacting pixels. It should be noted that, based on previous bending test results, the GI pressure sensor exhibits significant sensitivity differences under varying bending radius. Therefore, calibration is necessary when the sensor is used on complex curved surfaces such as body attachment.

Precise synergistic touch feedback capability is the key to fine‐tuning the human hand. GI pressure sensor have ultra‐high sensitivity, and we demonstrate a bionic hand that is integrated into our GI devices into the five fingertips to enable the manipulator to capture dynamic stimuli and provide feedback for closed‐loop control. As shown in Figure 6E, the bionic manipulator with five GI sensors integrated into the fingertips in a conformal manner enables the acquisition of multi‐channel capacitive signals. At the same time, the sensors do not interfere with the free bending and deformation of the manipulator. When a human finger touches the bionic manipulator and interacts with it, the GI pressure sensors at the corresponding positions of the manipulator can generate measurable capacitive signal changes (Figure 6E,F). Also, when we used a tennis ball to roll over the four fingers of the bionic robot hand, the change in capacitance of the GI sensors on each finger throughout the dynamic process can be continuously capture (Figure 6G). Sensors that do not come into mechanical contact with the outside environment during testing maintain signal stability.

An interactive robotic hand with close‐loop feedback control is demonstrated by co‐conformally attaching two GI pressure sensors to the thumb and index finger of a bionic robotic hand. The sensor on the index finger is used to detect the force used when grasping an object to ensure that the force is at the right magnitude to not cause destructive damage to the object. The two sensors on the thumb and index finger work together to monitor that the robotic hand is operating within safety thresholds to avoid damage to the devices. The rotational bending of the robot fingers is controlled by servo motors mounted inside the robot hand. By using a proportion‐integration‐differentiation (PID) controller, the bionic robot hand can be realized to dexterously manipulate precision objects by utilizing the capacitive signal feedback from the sensors mounted at the fingertip positions, and its feedback control working schematic is shown in Figure 6H. A safety threshold C_T_ for the pressure sensors is set to prevent damage to the device caused by the uncontrolled grip of the bionic fingers in the event of an accident. θ_0_ is the rotation angel of the servomotor when the five fingers of the robot are naturally open without bending. After the system is activated, a balloon is placed between the thumb and index finger of the bionic robot hand, and the motors control the finger joints to undergo bending and rotation in order to grasp the balloon. When the bionic machine finger touches the balloon, the capacitance value (C_2_) of the GI pressure sensor on the tip of the index finger increases rapidly. C_2preset_ is the capacitance value required by the sensor on the index finger corresponding to a correct grip on the target. The actual measured value of C_2_ of the GI pressure sensor on the index finger is fed back to the PID controller in real‐time, and the difference between C_2_ and C_2preset_ is calculated by the PID to output the rotational angle required for the motor to drive the bionic robot to bend its finger. The whole iterative control process can be revealed from the fluctuation of the pressure on the index finger near the target pressure in Figure 6I (stage 1 to stage 3). After the feedback closed‐loop control is completed, the robot hand can realize the thumb and index finger gently grasping the balloon with appropriate force. At this time, the balloon does not fall and is not deformed or even destroyed because of the excessive grip force. By repeatedly inflating and deflating the balloon through the air pump, the bionic robot hand can follow the expansion and contraction of the balloon to adjust the bending angle of the fingers to ensure that the fingers can always keep gently grasping the balloon (as shown in Video S1, Supporting Information).

## 3 Conclusion

We present an iontronic pressure sensor with ultrahigh sensitivity and a tunable linear pressure range constructed based on a material modulus tuning mechanism. The Young's modulus of the electrode can be effectively changed by tuning the content of silver nanowires in the self‐assembled 3D porous graphene. Electrode materials with small moduli are more likely to deform under external compression thus causing a significant increase in the EDL capacitance of the iontronic pressure sensor. However, the pressure threshold corresponding to its response saturation threshold tends to be lower as well. By controllable modulation of the electrode modulus, we realize an ultra‐high sensitivity of 25 548.24 kPa^−1^ and a wide adjustable linear pressure response range of 127 kPa. And the device can feel a tiny pressure of 0.24 Pa and maintain good stability during 10 000 cycles of 7 kPa pressure loading/unloading. Our investigation, based on the relationship between the material modulus and the performance modulation of ionic electronic pressure sensors, provides ideas for the development of high‐performance pressure sensors. We anticipate that the modulus‐modulation strategy can be widely extended to many other devices.

## 4 Experimental Section

### Materials

Polyvinyl alcohol (PVA, Macklin), Dimethyl sulfoxide (DMSO, Macklin), Sodium chloride (NaCl,), Oxidized graphene dispersion (GO, XFNANO), Sodium ascorbate (C_6_H_7_NaO_6_, Aladdin), Conductive PI film coated with copper foil (PI‐Cu, Dianjin New Materials)

### Fabrication of 3D Porous Graphene

Before preparation, the graphene oxide dispersion needs to be pretreated to achieve the desired concentration and uniformity. First, centrifuge the solution at 3000 r min^−1^ for 30 min and collect the supernatant. Then, centrifuge the supernatant again at 8000 r min^−1^ for 30 min and collect the precipitate. Finally, sonicate the precipitate to obtain a uniform graphene oxide dispersion and calibrate its concentration to 2.5 mg mL^−1^. Uniformly dispersed graphene oxide (2.5 mg mL^−1^) and C_6_H_7_NaO_6_ with a mass ratio of 1:1 were added into a glass vial. 10, 20 wt.% silver nanowires were also added for 10%AgNW‐Graphene, 20%AgNW‐Graphene samples, respectively. The above solutions were homogeneously mixed and sealed in an oven at 95 °C for a full reaction of 6 h. During this process graphene oxide was reduced and self‐assembled to form a 3D structure. Wash with deionized water for 3–5 times to remove the residual C_6_H_7_NaO_6_. 3D porous graphene electrodes were obtained after freeze–drying for 8–10 h.

### Fabrication of Conical Hydrogels

Microconical arrays of Teflon plates with a base diameter of 0.9 mm and a depth of 0.4 mm were carved using a flat‐bottomed sharp knife (angle 90°) as templates for the preparation of conical hydrogels. The hydrogels were prepared by the solvent displacement method. PVA (2 g) was dissolved in DMSO (10 mL) and stirred at 95 °C for 2 h. The resulting clear solution (2 mL) was poured into the conical molds. After vacuum defoaming, the gel was formed by immersion in deionized water at room temperature for 48 h. The deionized water was replaced every 6 h, with a final soaking in 1 mol L^−1^ NaCl saline solution and ensuring that the DMSO was thoroughly replaced.

### Fabrication of Porous PDMS

The polydimethylsiloxane (PDMS) matrix and the curing agent (10:1 mass ratio) were mixed well, vacuum degassed, and spin‐coated on the surface of the sandpaper template at 150 r min^−1^. After curing at 80 °C for 1 h, the PDMS film with microstructure (thickness ≈0.35 mm) was peeled off. A laser engraving machine was used to prepare a porous array with bottom pore size of ≈0.35 mm, top pore size of ≈0.26 mm, and a pore spacing of ≈1.3 mm on the PDMS film.

### Fabrication of GI Pressure Sensor

The PDMS film with a porous array was placed on the conical hydrogel. Each hole and cone were matched one by one. Two matched films were sandwiched between the porous graphene electrode and the PI–Cu. The leads were bonded using silver paste. Finally, polyimide (PI) tape is used for encapsulation.

### Characterization

The morphology of the porous graphene electrode was observed by field emission scanning electron microscopy (GeminiSEM 300, ZEISS). Porous graphene‐specific surface area and pore size distribution results were obtained from surface adsorption analyzer detection (ASAP 2020 Plus). Microstructure of hydrogel active layer and PDMS spacer layer observed by charge‐coupled device (A0‐FD600E, AOSVI). An LCR meter (E4980AL, KEYSIGHI) was used to measure the capacitance of the sensors. The applied pressure was controlled and recorded by a tensile measurement machine (LD23.501, LSD).

### Finite Element Analysis

The FEA was performed using the ABAQUS 2020. The porous graphene model was constructed using the Thiessen polygon that is widely used in academia. Graphene imparts a materials density of 2.2 × 10^3^ kg m^−3^ and a Young's modulus of 50 GPa. The porous graphene model was constructed with a Youns's modulus of 2.27 kPa. The error in Young's modulus with respect to the measured Young's modulus of the experimental sample is less than 5%.

### Experiments on Human Subjects

All experiments were conducted under approval from the Institutional Review Board at the Southern University of Science and Technology under protocol number: 20 210 138.

## Conflict of Interest

The authors declare no conflict of interest.

## Supporting information

Supporting Information

Supplemental Video 1

## Data Availability

The data that support the findings of this study are available in the supplementary material of this article.
