# Supplementary material for: Pushing Pressure Detection Sensitivity to New Limits by Modulus‐Tunable Mechanism
Source: Adv Sci (Weinh). 2024 Jul 8;11(34):2403779. doi: 10.1002/advs.202403779 (PMC11425887; doi:10.1002/advs.202403779)
Supplement: Supplementary file 1 — Supporting Information [file ADVS-11-2403779-s002.docx]

Supporting Information

Pushing Pressure Detection Sensitivity to New Limits by Modulus-Tunable Mechanism

Jing Yang^1^, Guojiang Yuan^1^, Yong Shen^1^, Caili Guo^3^, Zhibin Li^1^, Fengling Yan^1^, Xiaolong Chen^1, 2,^ *, Lin Mei^3,^ *, Taihong Wang^1, 2,^ *

**
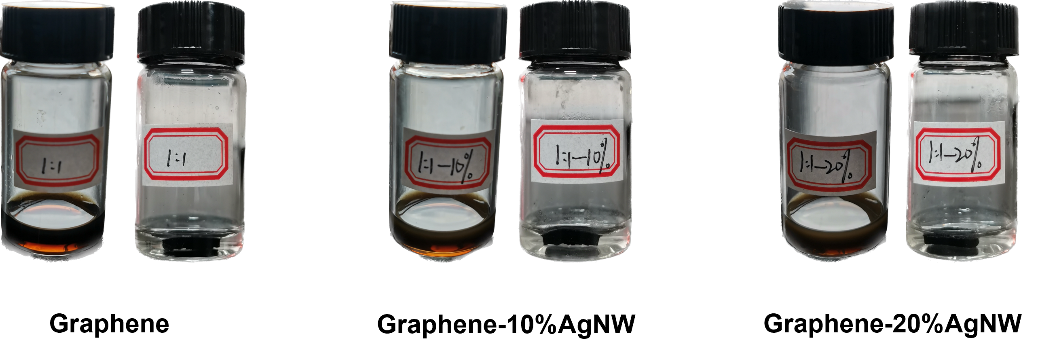
**

**Figure S1. Optical photographs of the self-assembly process of three-dimensional porous graphene.** From left to right, the comparative images of the reductive self-assembly of graphene oxide with 0%, 10%, and 20% AgNW content into three-dimensional porous graphene.

**
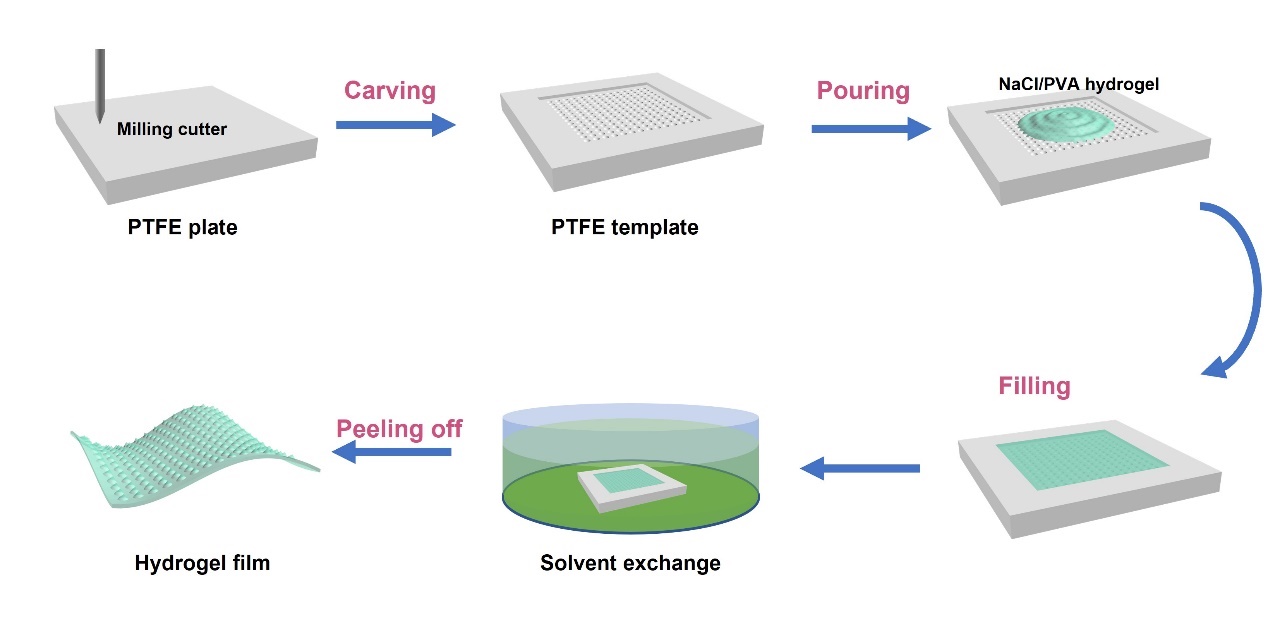
**

**Figure S2. Process for the preparation of NaCl-PVA hydrogels with microcone array microstructures.**

**
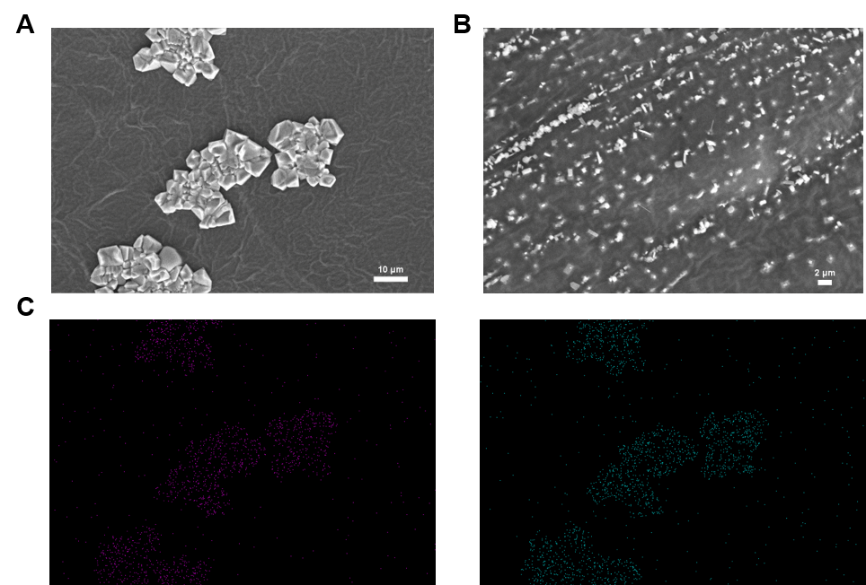
**

**Figure S3. Characterization results of the geometric morphology and elemental distribution of the NaCl-PVA hydrogel.** A) SEM image the NaCl-PVA hydrogel after freeze-drying; B) SEM image of the cross-section of the NaCl-PVA hydrogel after freeze-drying; C) Energy-dispersive X-ray spectroscopy (EDX) elemental maps of Na and Cl elements on the surface of the NaCl-PVA hydrogel after freeze-drying (corresponding to the SEM image in A).

**
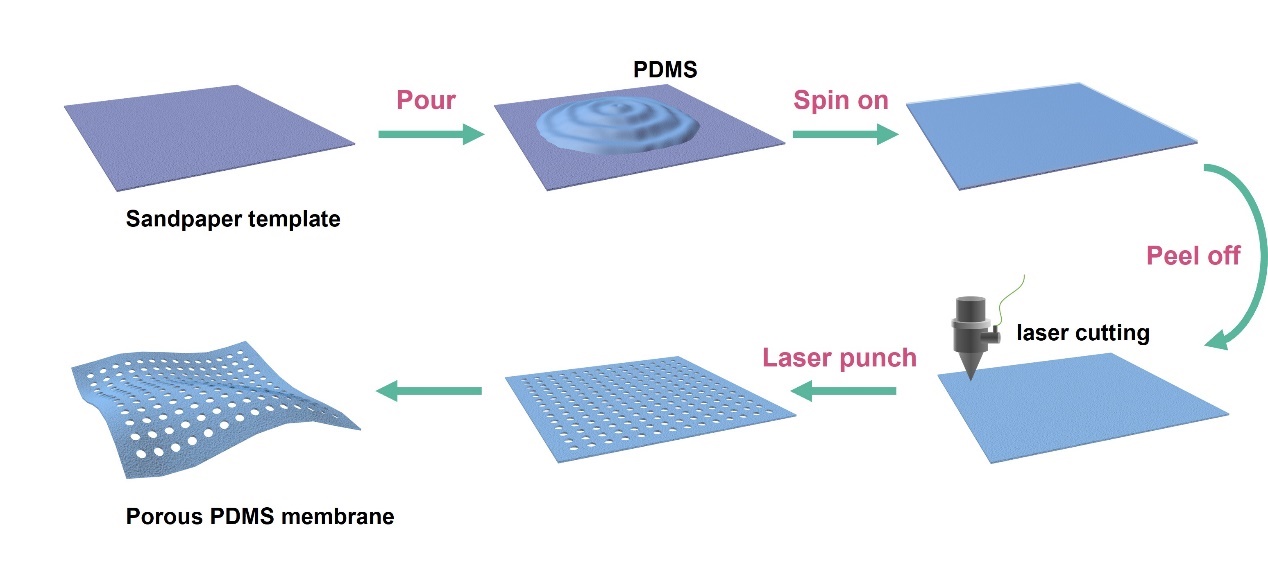
**

**Figure S4. Schematic diagram of the production process for through-hole PDMS spacer.**

**
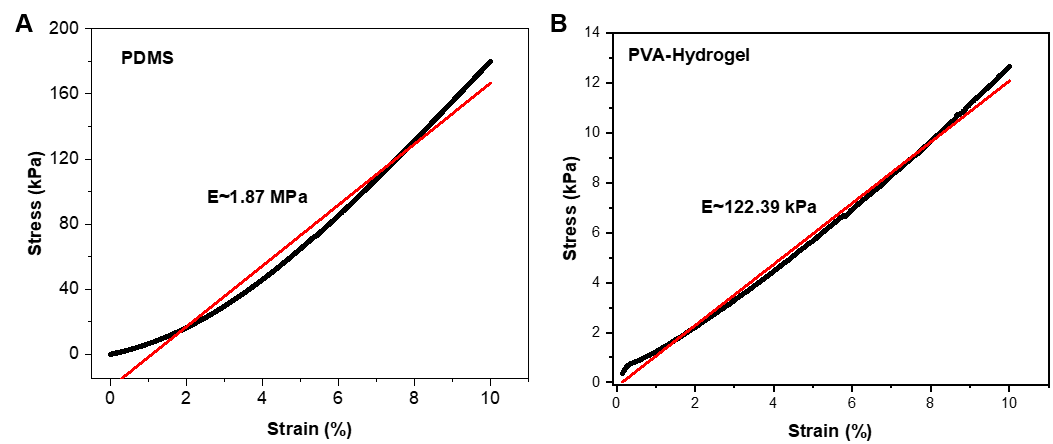
**

**Figure S5. compressive stress-strain curve.** A) PDMS; B) NaCl-PVA hydrogel.

**
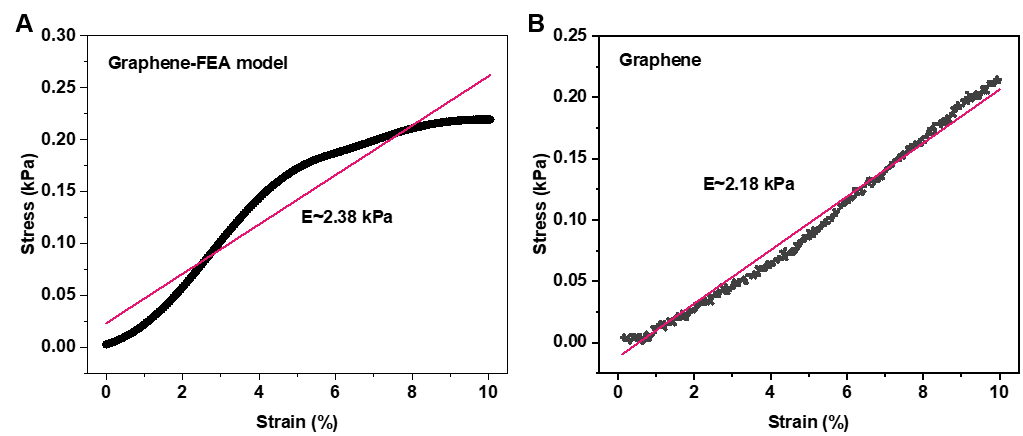
**

**Figure S6. Compressive stress-strain curves.** A) Simulation results of the Young's modulus for the three-dimensional porous graphene model. B) Compressive stress-strain curves for pure graphene electrode. The corresponding scatter in each figure is the experimental data and the red line is the fitting result.

**
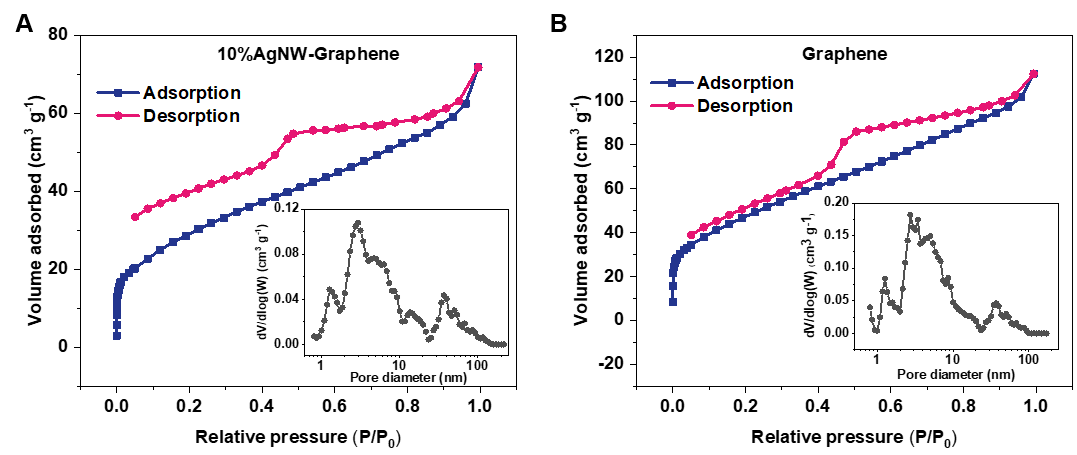
**

**Figure S7. Nitrogen adsorption/desorption analysis of 3D porous graphene with different silver nanowire contents.** A) Graphene electrode with 10%wt AgNW content. B) Pure graphene electrode.

**
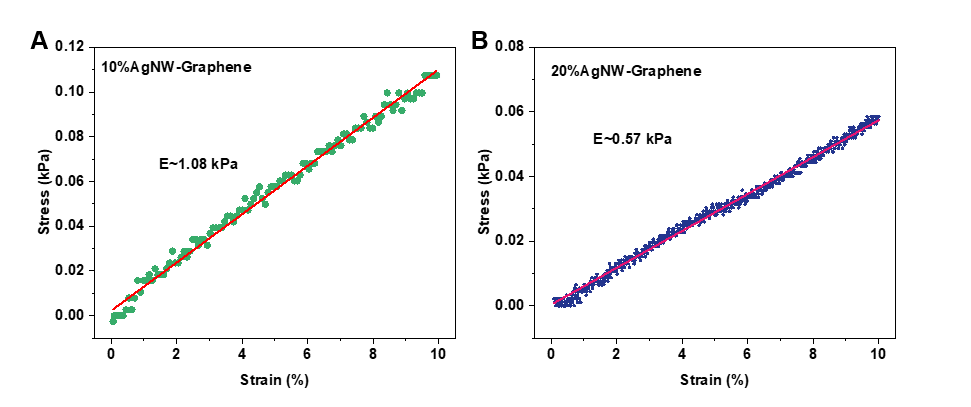
**

**Figure S8. Compressive stress-strain curves.** A) Graphene electrode with 10%wt AgNW content. B) Graphene electrode with 20%wt AgNW content. The corresponding scatter in each figure is the experimental data and the red line is the fitting result.

**
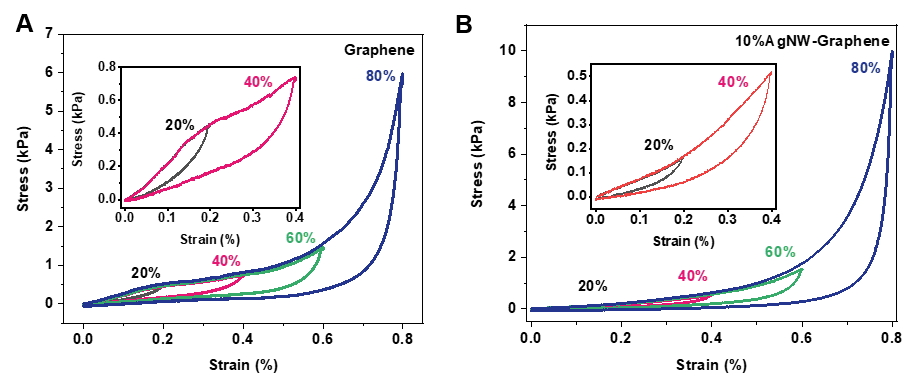
**

**Figure S9. Compressive stress–strain curves during compression-release cycles with maximum strains up to 80%.** A) Pure graphene electrode. B) Graphene electrode with 10%wt AgNW content.

**
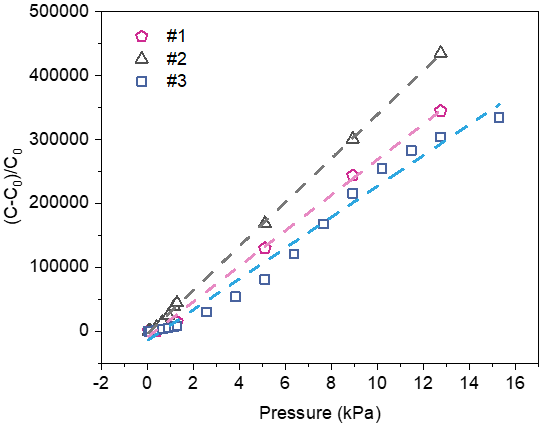
**

**Figure S10. Sensitivity comparison of pressure sensors manufactured in different batches.** The comparison of capacitance normalization curves for 20A-G pressure sensors made in different batches.

**
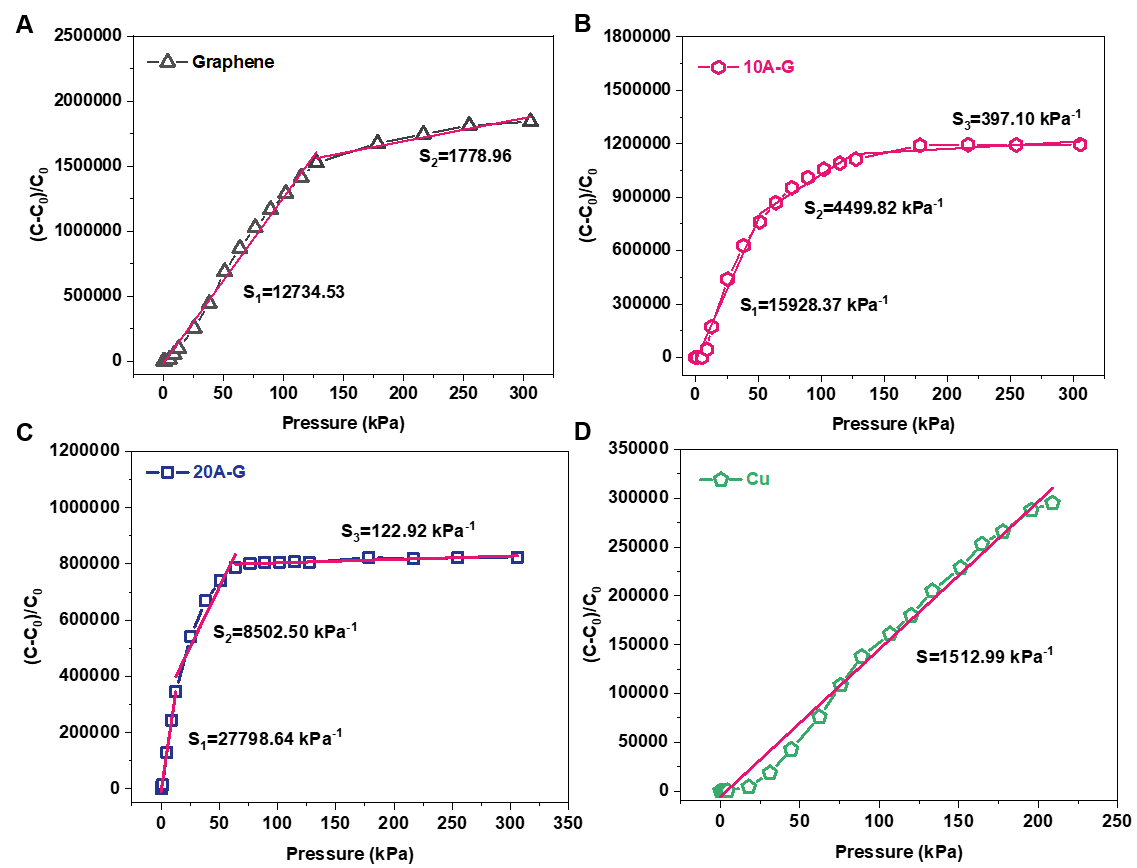
**

**Figure S11. Normalized change in capacitance as a function of the pressure for the prepared ionic pressure sensor with different electrode.** A) ionic pressure sensor prepared by pure graphene electrode. B) ionic pressure sensor prepared by graphene electrode with 10%wt AgNW content. C) ionic pressure sensor prepared by graphene electrode with 20%wt AgNW content. D) ionic pressure sensor prepared by Cu electrode.

**
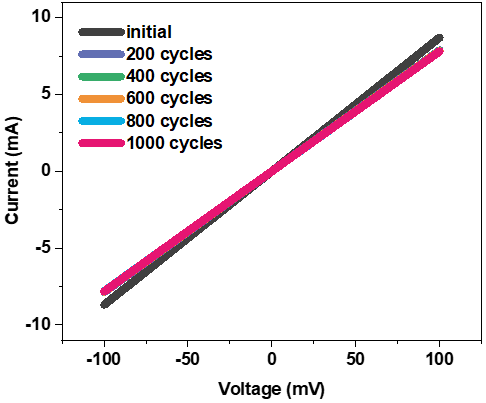
**

**Figure S12. The I-V measurement curves of the 3D porous graphene electrode containing 20% AgNW after different numbers of loading and unloading cycles at a peak stress of 7 kPa.**

**
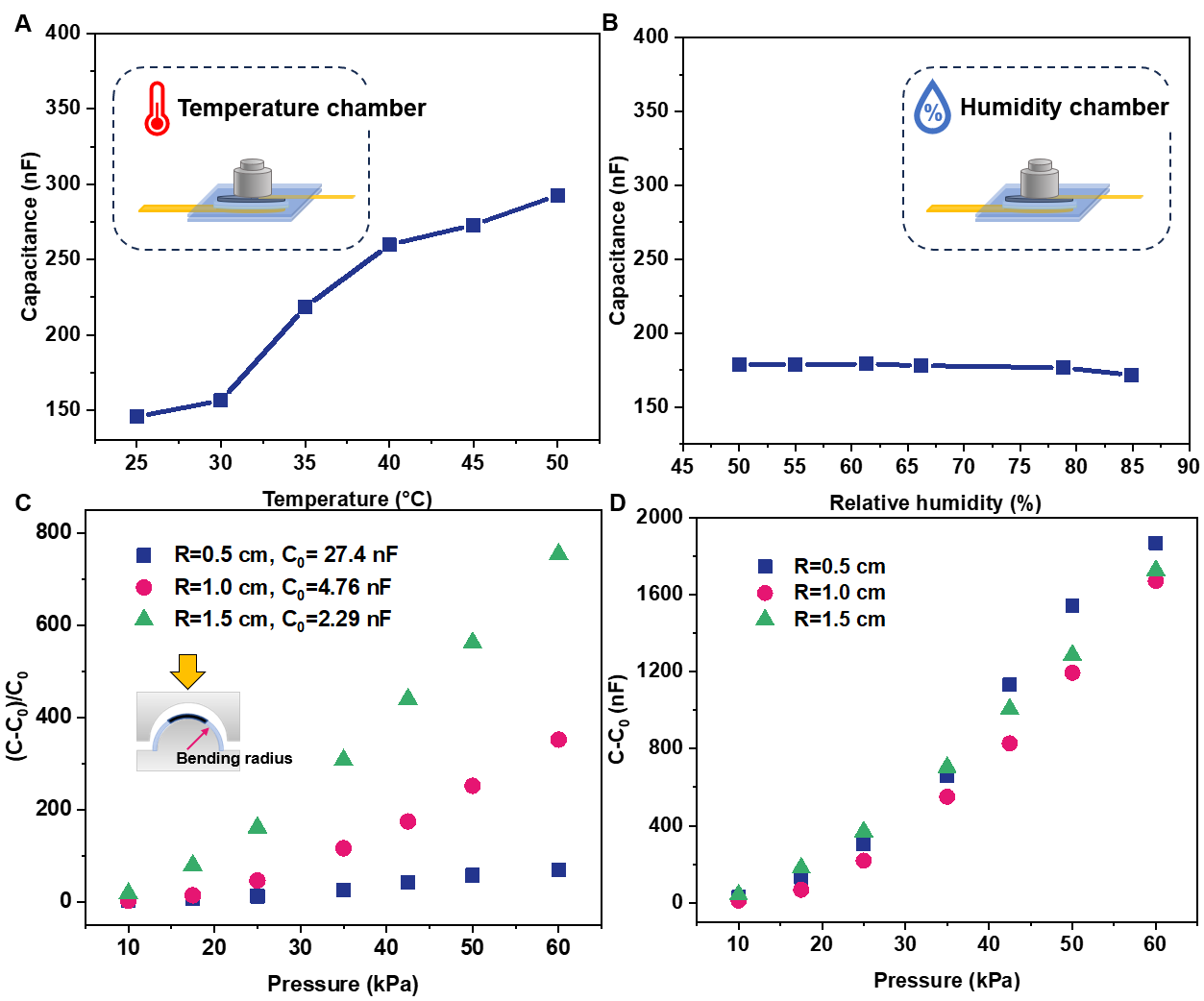
**

**Figure S13. Performance variations of the GI pressure sensor under environmental changes and mechanical deformation.** When a 20 g fixed mass weight is placed on the surface of the GI sensor, A) the change in capacitance of the sensor within the ambient temperature range of 25°C to 50°C; B) The change in capacitance of the sensor within the ambient humidity range of 50% to 85%; C) The normalized capacitance of the sensor as a function of pressure under different bending radius; D) The change in capacitance (ΔC=C-C_0_) of the sensor as a function of pressure under different bending radius.

**Table S1. Comparison of the sensitivity and the operating range between the 20%AgNW-Graphene pressure sensor and capacitance sensors previously reported.**

| **Device type** | **Sensitivity [kPa^-1^]** | **LOD** | **Pressure range** | **Linearity range (at the highest sensitivity)** | **Response/**  **Recovery time** | **References** |
| --- | --- | --- | --- | --- | --- | --- |
| Iontronic | 42.64 (0-1.1 kPa) 230.10 (1.1-100 kPa) | 6.8 Pa | 6.8 Pa-100 kPa | 1.1 kPa-100 kPa (230.10) | 258 ms | 32 |
| Iontronic | 32.7 (0.04 Pa-209 kPa) | 0.04 Pa | 0.04 Pa-209 kPa | 0.04-209 kPa (32.7) | 64 /73 ms | 20 |
| Iontronic | 3302.9 (0.06 Pa-10 kPa) 671.7 (10-100 kPa) 229.9 (100-360 kPa) | 0.08 Pa | 0.08-360 kPa | 0.08 Pa-10 kPa (3302.9) | 9 /18 ms | 33 |
| Iontronic | 0.24 (0-70 kPa) 1.5 (70-150 kPa) 0.13 (150-330 kPa) | 35 Pa | 35 Pa-330 kPa | 70-150 kPa (1.5) | 18 / 36 ms | 34 |
| Iontronic | 17.8 (0-60 kPa) 73.3 (60-100 kPa) 33.8 (100-150 kPa) | 5 Pa | 5 Pa-150 kPa | 60-100 kPa (73.3) | 11/ 8 ms | 36 |
| Iontronic | 55.66 (0-31.11 kPa) 24.72 (31.11-66.67 kPa) | 0.087 Pa | 0.087 Pa-66.67 kPa | 0-31.11 kPa (55.66) | 111.70 / 158.65 ms | 37 |
| Iontronic | 9280 (2-20 kPa) 1811.4 (20-60 kPa) 627.7 (60-114 kPa) | / | 2-114 kPa | 2-20 kPa (9280) | 10 / 28 ms | 23 |
| Iontronic | 185.09 (0.49-66.67 kPa) | 0.49 Pa | 0.49 Pa-66.67 kPa | 0.49 Pa-66.67 kPa (185.09) | 60 / 60 ms | 38 |
| Iontronic | 10420.8 (0–12.5 kPa) 4536.2 (12.5-80 kPa) 1439.8 (80-300 kPa) | 0.2 Pa | 0.2 Pa-300 kPa | 0.2 Pa-12.5 kPa (10420.8) | 40 / 35 ms | 39 |
| Iontronic | 13786.2 (0-10 kPa) 3562.1 (10-60 kPa) 208.9 (60-300 kPa) | 0.1 Pa | 0.1 Pa-300 kPa | 0.1 Pa-10 kPa (13786.2) | 8 / 16 ms | 40 |
| Iontronic | 6052.37 (<0.6 kPa) 25548.24 (0.6-15 kPa) 8502.5 (15-64 kPa) 122.92 (64-306 kPa) | 0.24 Pa | 0.24 Pa-306 kPa | 0.6 kPa-15 kPa (25548.24) | 47.79 /57.34 ms | This Work  (20A-G) |
| Iontronic | 12734.53 (< 127 kPa) 1778.96 (127-306 kPa) | 0.24Pa | 0.24 Pa-306 kPa | 0.24 Pa-127 kPa (12734.53) | / | This Work  (0A-G) |
